# Correcting non cephalic presentation with moxibustion: study protocol for a multi-centre randomised controlled trial in general practice

**DOI:** 10.1186/1472-6882-8-22

**Published:** 2008-05-21

**Authors:** Jorge Vas, José Manuel Aranda, Mercedes Barón, Emilio Perea-Milla, Camila Méndez, Carmen Ramírez, Inmaculada Aguilar, Manuela Modesto, Ana María Lara, Francisco Martos, Antonio J García-Ruiz

**Affiliations:** 1 Pain Treatment Unit, Primary Healthcare Centre, Dos Hermanas, Spain; 2 San Andrés-Torcal Primary Healthcare Centre, Málaga, Spain; 3 El Lugar Primary Healthcare Centre, Chiclana de la Frontera, Spain; 4 Support Research Unit (Network and Cooperative Research Centres of Epidemiology. CIBERESP), Costa del Sol Hospital, Marbella, Spain; 5 Andalusian Public Health System, Sevilla, Spain; 6 Doña Mercedes Primary Healthcare Centre, Dos Hermanas, Spain; 7 Gonzalo Bilbao, Primary Healthcare Centre, Sevilla, Spain; 8 Department of Pharmacology, Malaga University, Spain

## Abstract

**Background:**

Non cephalic presentation in childbirth involves various risks to both the mother and the foetus. The incidence in Spain is 3.8% of all full-term pregnancies. The most common technique used to end the gestation in cases of non cephalic presentation is that of caesarian section, and although it provokes a lower rate of morbi-mortality than does vaginal delivery in such situations, there remains the possibility of traumatic injury to the foetal head and neck, while maternal morbidity is also increased. The application of heat (moxibustion) to an acupuncture point, in order to correct non cephalic presentation, has been practised in China since ancient times, but as yet there is insufficient evidence of its real effectiveness.

**Methods/Design:**

The experimental design consists of a multi-centre randomised controlled trial with three parallel arms, used to compare real moxibustion, sham moxibustion and the natural course of events, among pregnant women with a non cephalic presentation and a gestational duration of 33–35 weeks (estimated by echography). The participants in the trial will be blinded to both interventions. The results obtained will be analyzed by professionals, blinded with respect to the allocation to the different types of intervention. In addition, we intend to carry out a economic analysis.

**Discussion:**

This trial will contribute to the development of evidence concerning moxibustion in the correction of non cephalic presentations. The primary outcome variable is the proportion of cephalic presentations at term. As secondary outcomes, we will evaluate the proportion of cephalic presentations at week 38 of gestation, determined by echography, together with the safety of the technique, the specificity of moxibustion and the control of the blinding process.

This study has been funded by the Health Ministry of the Andalusian Regional Government.

**Trial registration:**

Current Controlled Trials ISRCTN10634508.

## Background

Non cephalic presentation (NCP) in childbirth involves various risks to both the mother and the foetus. NCP includes breech, oblique and transverse presentations. The incidence of breech presentation (BP) is 20% at 28 weeks' gestation, although spontaneous version tends to occur more frequently [1], such that in only 3–4% of women with a full-term pregnancy is the foetus BP [2]; in Spain, NCP represents 3.8% of all births [3].

Until week 28–32, and under normal conditions, the foetus has a high degree of mobility, and remains in an unstable situation. From the latter date, the foetus adapts itself to the shape of the uterus, adopting a head-down posture, and normally remains so until birth. There are various reasons that may prevent the foetus from moving into the cephalic position, the most common of which, according to a specialized study [4] are: a) pre-term birth; this factor is more influential with lower gestational ages (35% before week 28, 16.8% from week 28–31, 8.9% from week 32–36 and 3.7% from week 36–41); b) foetal malformations (those most commonly related to NCP are anencephaly, hydrocephaly, spina bifida, polycystic kidneys and Potter's Syndrome, as well as trisomies 13, 18 and 21; c) multiple pregnancy, in which the incidence of NCP is 7 times greater than in the case of a singleton pregnancy. High levels of perinatal mortality and morbidity are known to be associated with NCP, due mainly to prematurity, congenital malformations, hypoxia or birth trauma [5,6], and so a breech delivery is a birth that is categorized between dystocia and eutocia.

The most common technique used to end gestation in cases of NCP is that of caesarian section. From the results of the study by Hannah et al. [7], in October 2000, from the results produced by the Term Breech Trial Collaborative Group, and from the Cochrane Collaboration review [8], we now know that foetuses which are extracted by elective caesarian section have a lower level of perinatal morbi-mortality than those delivered vaginally. However, this technique does not prevent traumatic lesions to the foetal head and neck and, moreover, it is associated with an increase in maternal morbidity [8-11]. In addition to the increase in morbidity immediately following a caesarean section, there may occur other types of complications, such as intraabdominal adhesions, with the possibility of later infertility and risks in future pregnancies caused by the uterine scar [11].

Due to the risks arising both in breech presentation and from a caesarean section, various manoeuvres have been suggested over the years to promote spontaneous cephalic version of the foetus during the final two months of the pregnancy. Of these actions, the most commonly adopted is that of external cephalic version (ECV), which can be performed either between week 32–34 of gestation, or after week 37. A Cochrane Review concluded that, in comparison with the non-performance of ECV, the latter technique, when begun pre-term, reduces NCP births, while, in comparison with full-term ECV, beginning it between week 34–35 may be of some benefit as concerns the reduction of the rates of NCP and of caesarean section, although further trials were recommended [8]. Other types of manoeuvres and techniques have been tested, with varying degrees of success, in order to increase foetal mobility in a non-traumatic way.

Acupuncture and moxibustion are therapeutic techniques found in traditional Chinese medicine. They have been employed since ancient times, and specific recommendations in Chinese texts include the application of heat (moxibustion) by the combustion of *Artemisia vulgaris *(moxa) over an acupuncture point in order to correct NCP [12,13]. This latter point, known as *Zhiyin *(BL67) is located in the outer corner of the little toenail. In addition to moxibustion, acupuncture may also be used, either alone or in association with moxibustion, on this or other points, although it has been found that the highest level of effectiveness is associated with the application of moxibustion alone [14]. A Cochrane review [15] concluded that there is insufficient evidence from the studies included in its analysis, and observed that there have been few well-designed studies (only 3 are included in the review) and that most of them used a small sample. Some studies with a larger sample, such as that by Kanakura et al. [16], were not randomized. A larger, systematic review that we have carried out this year (manuscript submitted for publication), with the inclusion of new studies performed to date, and with the incorporation of those published in Chinese in the last 10 years, shows that studies contain to be of low methodological quality. We estimate a correction rate of 70% (versus 50% in control groups) among pregnant women treated with moxibustion. Among the studies carried out so far, no reports have been made of side effects, although the smoke generated while the moxa is burned may irritate the respiratory tracts. Nevertheless, there is no evidence to support this hypothesis [15]. Neri et al. [14,17] have made a cardiotocographic study of the effects of moxibustion on foetal heartbeat, and concluded that no harm is caused by this process.

The present study is funded by the Health Ministry of the Andalusian Regional Government (Project No. 0053/2007).

## Methods

### Research aims and questions

The aim of this study is to assess the effects of sensorial stimulation with moxibustion in the outer corner of the little toenail (BL67) in order to produce version in non-cephalic presentations.

### Research questions are

How effective is moxibustion at point BL67 in terms of the rate of full-term cephalic presentations?

How effective is moxibustion at point BL67 in terms of the rate of caesarean sections?

What is the effect of moxibustion at a non-specific point such as SP1 in pregnancies with NCP?

To what extent are the pregnant women to whom the study techniques are applied confident of the outcome?

How safe is the technique in terms of increased rates of changed foetal cardiac rhythm, the risk of premature birth, and changes in APGAR scores at 1 minute and at 5 minutes?

What is the cost-effectiveness of treatment with moxibustion for cases of NCP?

### Duration of Study

April 2008 to December 2010

### Subjects

Pregnant women recruited by their obstetrician during the third scheduled echography (at week 32 of gestation), after confirmation of NCP. These pregnant women will be referred to midwives at the participating Primary Healthcare Clinics belonging to the Andalusian Public Health System in the provinces of Sevilla, Huelva, Cádiz and Málaga. The pregnant women will be informed as follows: "If you decide to take part in the study, in addition to being given various postural recommendations, you will be included, at random, in one of the three treatment groups. In two of these, moxibustion techniques will be applied at a specific point on each foot, while in the third group the standard treatment procedures followed in the healthcare system will be applied, to see if spontaneous correction occurs. If you are assigned to one of the two groups in which moxibustion is applied, then, according to the random choice made, you may be given moxibustion at a point that has been proved to be effective, or on the contrary, at a point that has not proved to be of any value for the aims of our study". The pregnant women will also be informed of the possible risks associated with the use of moxibustion (respiratory discomfort from the smoke inhaled, the possibility of burns or of ulceration of the skin) and that they may conclude their participation in the study at any time, with no type of penalty or loss of benefits they would otherwise be entitled to.

The subjects included in the study will be pregnant women, aged at least 18 years, whose foetus is in a non cephalic position (diagnosed by physical examination and by echography), with a gestational age of 33–35 weeks (estimated by echography), normal foetal biometry, who have signed their informed consent and who have not previously received moxibustion treatment to correct the position of their unborn baby. Criteria for exclusion will include twin pregnancy, pelvic defect, previous uterine surgery, foetal malformation or chromosome disorder, uterine malformation, risk of premature birth (preterm uterine contractions and/or initial dilation or shortening of the cervix with a Bishop's score of 4), uterine fibromas > 4 cm, treatment with tocolytic drugs, heart or kidney disease affecting the mother, other pregnancy-related complications, or incapacity to complete the questionnaire or respond to the assessor's questions.

The ethical validity of this study has been analysed and approved by the Clinical Trials Ethics Committee of the Andalusian Regional Government, following approval by the corresponding Research Committee at each of the participating clinics.

### Randomization and treatment allocation

The randomization of the three branches of the study will be carried out on a centralized basis, by a statistician who is otherwise unrelated to the study, using a 1:1:1 scheme in blocks of 12 and stratifying by clinics. There will be the same number of sets of envelopes as there are of participating midwives/clinics (some 40 midwives are expected to take part). Each set will contain 12 opaque envelopes which will be sealed after the introduction of a card with the letter corresponding to the allocation to the group as determined by the software-generated sequence: Group A (true moxibustion), Group B (sham moxibustion) and Group C (control). After the baseline assessment and the signing of informed consent, these sealed, opaque, numbered envelopes will be used in sequence to assign the study subjects to one of the three groups. The collaborating midwife will note on the card the pregnant woman number and that of the clinic. At the end of the study, the chief researcher will check each card against the original randomization list to confirm that all the subjects received the treatment that had been assigned to them. This method ensures that neither the clinics nor the healthcare professionals taking part in the study will be involved in the randomization process and thus will not be able to influence it. A patient may be withdrawn from the study at any time, at her discretion or that of the researcher. The reasons for this will be noted down on the summary page of the Data Collection Record (DCR). The midwives who participate in the study have attended a 10-hour training course on postural recommendations and moxibustion techniques, and in which criteria for data collection were standardised.

### Sample size

The sample size has been predetermined at n = 492 patients, for a significance level of 95%, a power of 90% and a difference in the success rates of either of the intervention groups (A or B) with the control group (C) of 20%, assuming a dropout rate of 25%. These values were calculated using SamplePower version 2 software.

### Interventions

All the study subjects will be given various postural recommendations.

**Figure 1:**
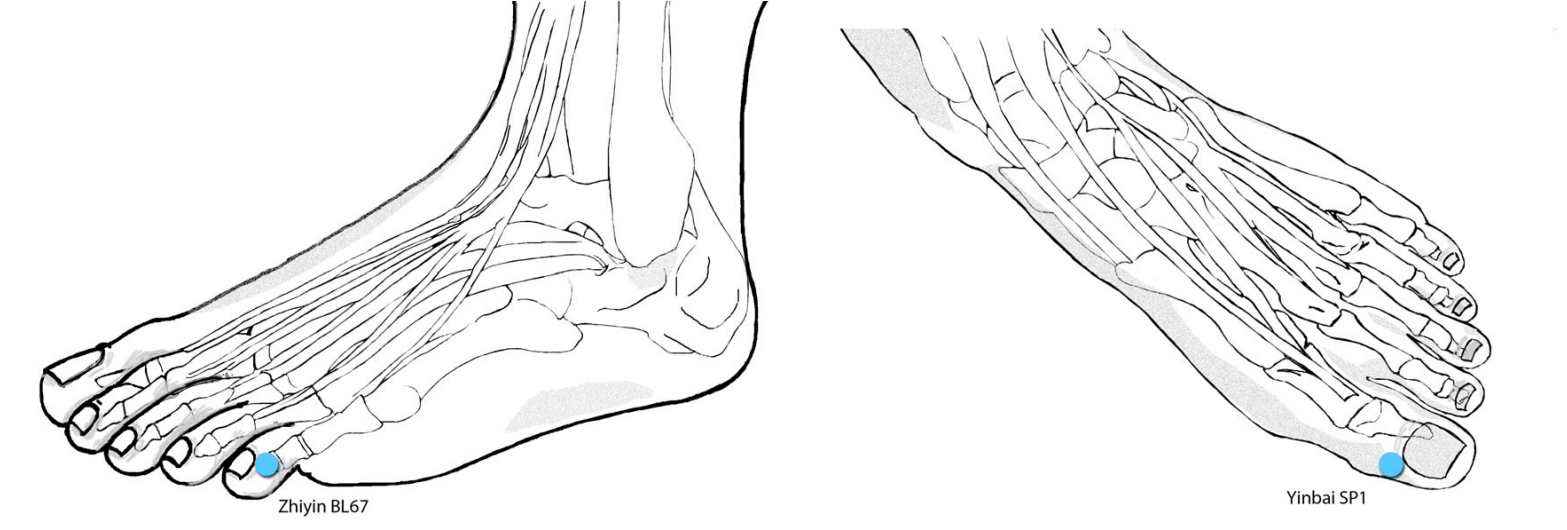
**Acupuncture points used**. Location of the two acupuncture points used in the study; Zhiyin BL67 for Group A and Yinbai SP1 for Group B.

True moxibustion (TM): The first intervention will be made at the Healthcare Clinic. This session (overseen by the midwife responsible) will serve as a training session for the friend or relative who is to apply the remaining sessions, at home. The mother should lie down, face up, wearing comfortable clothing and with no pressure at the waist from buttons, belt, etc. and with her legs slightly bent at the knee. Heat will be applied for 20 minutes, by means of a moxa cone (herbal preparation with *Artemisia vulgaris*) at point BL67 (*Zhiyin*), close to the outer angle of the little toenail (Figure 1). The heat will be applied from a distance of 1.5–3 cm, to ensure that the subject notices a feeling of warmth in the zone, but without any pain or burning. The application site (right or left foot) will be alternated every 2 minutes or more often if any untoward sensation of heat is felt.

False/Sham moxibustion (SM): This procedure will be identical to that applied to the TM group except that the point stimulated will be a non-active one – according to the principles of Traditional Chinese Medicine, point SP1 (*Yinbai*), close to the inner angle of the big toenail (Figure 1).

Control group, under observation (CG): The subjects in this group will receive the standard treatment, in accordance with the Andalusian Healthcare System Treatment Guide "*Embarazo, parto y puerperio*" (Pregnancy, birth and childcare) (2^nd ^edition).

### Outcome measures

#### Primary outcome

The rate of cephalic presentation at term will be taken as the primary endpoint variable.

#### Secondary outcomes

**Figure 2:**
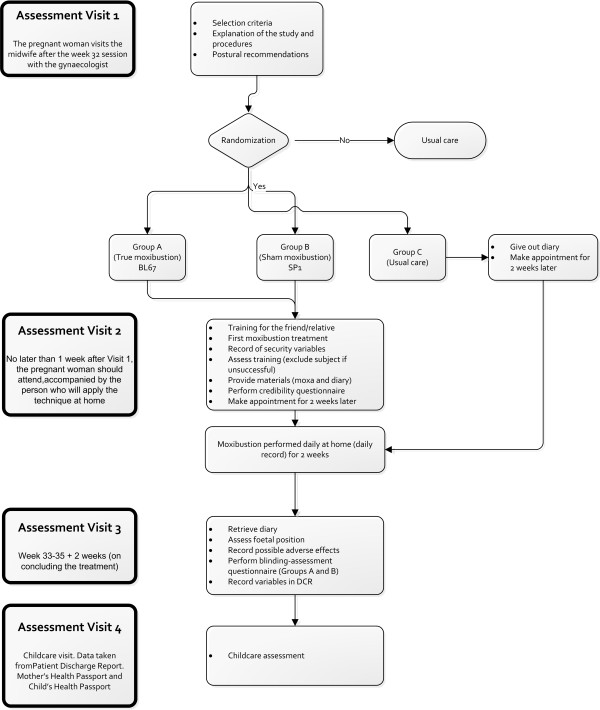
**Flow diagram for the study**. Work scheme with description of assessment visits and times.

Secondary outcomes will include the rate of cephalic presentation at week 38 of gestation (CP38) (determined by echography) and the number of days of treatment received until version occurs. The intervention allocation to the moxibustion groups (true or sham) will be controlled by asking the subject: "What type of treatment do you think you received?" Three possible answers may be given: A) Real; B) Sham; C) I don't know. The following sociodemographic covariables will be recorded: age, race, educational level, occupation, level of income, occupational activity, weight (kg), height (cm). In addition, these obstetric-gynaecological variables will be recorded: gestational age when subject was included in the study, determined by foetal biometry; type of presentation, determined by echography (characterized as breech, oblique or transverse presentation); foetal biometry (biparietal diameter, abdominal diameter and length of femur) [week of gestation when biometry is performed]; location of the placenta (categorized as anterolateral or posterior), parity (number of previous births), and previous miscarriages. The following security variables (in the first session) will be used: maternal heartbeat prior to the stimulus; foetal heartbeat prior to the stimulus; maternal heartbeat at 5 minutes after the stimulus; foetal heartbeat at 5 minutes after the stimulus. The security variables determined during the treatment will be recorded on the self-reported questionnaire which is completed by the subjects after each home treatment session, with data on foetal movements, uterine contractions, maternal heartbeat and any other incident following the stimulation. Finally, a number of other variables related to the birth will be recorded, including gestational age at birth, weight of the neonate, sex of the neonate, type of birth (vaginal or caesarean section), and APGAR score at 1 and 5 minutes (Figure 2).

### Data collection and analysis

#### Data collection

Most of the data obtained from the study will be recorded in the Pregnant Woman's Health Passport and in the Child's Health Passport. A Data Collection Record (DCR) form will be designed, including the variables of interest that will be noted down by the midwife collaborating with the healthcare clinic. We have designed a self-reported questionnaire for the subject to fill in after each session (during the two weeks' duration of the treatment), with data such as the number and intensity of foetal movements, the uterine contractions, maternal heartbeat and any relevant incidents that may have occurred following the stimulation. This information will be codified and sent weekly to a database for subsequent statistical analysis.

The baseline and intermediate evaluations, together with the results data obtained at birth, will be made by the corresponding midwife. The self-reported questionnaires will be oriented by the same evaluating personnel. Collateral effects and possible adverse reactions to the treatment will also be noted.

#### Statistical analysis

In the first place, we will perform an analysis following intention-to-treat principles, including all the subjects with valid data, irrespective of whether or not they received the treatment assigned. These results will be compared with those obtained by an on treatment analysis, in order to determine whether violations of the protocol led to bias. The subjects for whom deviations from the protocol are observed – such as not having received the complete treatment, or as having received insufficient follow up, will be excluded from the per treatment analysis. A comparison will be made of respondents and non-respondents to reveal the existence of any selection or classification bias.

Comparability between the interventions and usual care groups will be assessed at baseline to evaluate the differences. The magnitude of the difference in any imbalance produced by the random assignation to the groups will be evaluated using means and proportions ratios (using that of the usual care group as the reference level).

In the bivariate analysis of the magnitude of the association between exposure and effect, diverse tests of statistical significance will be employed to make comparisons between *k *samples (parametric or otherwise, depending on the asymmetric or otherwise distribution of the results variables and on the homogeneity of their variances), taking the usual care group as the reference level and using comparison tests for differences in proportions in the primary outcome measure.

For the primary outcome variable, logistic regression models will be created, using ITT analysis. These will include, firstly, the group variables (experimental vs control), taking the control group (usual care) as the reference level. Then, the model will be adjusted for the variables of age, parity and weeks of gestation at randomization (the baseline variables). Adjustment will also be made for other possible confounders (the variables that are imbalanced in the baseline analysis) using criteria of statistical significance (*P *< 0.05) and of confusion (change of over 10% according to the magnitude estimator – the incidence odds ratio). The detection of possible interactions with the "treatment group" variable will be carried out by means of statistical significance criteria for the corresponding terms of interaction, introducing the relevant additive and multiplicative interaction variables. The level of significance will be set at <0.05 α for the global comparisons, although this level will be penalized by the existence of an intermediate level, as explained below. A cost effectiveness analysis, from the standpoint of the Andalusian Public Healthcare System, will be performed.

#### Intermediate analysis

An intermediate analysis will be performed when the follow up of half of the total sample has concluded and the results of this are known, using multiple Bonferroni tests, and establishing a level of statistical significance for 2 tests at *P *< 0.025. This penalization is necessary to avoid exaggeration of any type I error.

## Discussion

As observed above, the problem identified by the analysts of the only Cochrane review available is the low methodological quality of most of the studies that have been made. In designing the present study, special care was taken regarding the characteristics conforming a quality RCT, in accordance with the recommendations made by Jadad [18]. Nevertheless, the following foreseeable limitations should be pointed out: firstly, the sample was extracted from the universe of pregnancies managed within the Andalusian Public Healthcare System (SSPA), and therefore the conclusions drawn will only be applicable to this population group. The complexity of including private clinics in the study led us to renounce this idea, especially in view of the fact that the coverage of the pregnancy control programme within the public system is almost universal; moreover, the pregnant women who make use of private systems do not stop receiving the follow-up attention scheduled under the public healthcare system. From the bibliographic review performed, neither does it seem that social class influences the incidence of breech presentation or its version during gestation. It is very likely, therefore, that the results obtained will resemble those that would be derived for the entire population of pregnant women in Andalusia. The inclusion of a routine control of the official programme (an echography performed in week 32) also avoids possible selection bias. Thus, with the involvement of the gynaecologist performing this examination, and of the midwife who participates in routine care, the uptake is ensured of 100% of the cases of NCP detected during the study period, while the rate of losses to follow up is minimised.

Secondly, there exists the possibility (which has actually occurred in other studies) of a pregnant woman refusing to take part when she discovers which branch of the research study she has been assigned to. This is applicable to the formation of the control group, as normally any intervention (whether true or sham moxibustion) will be willingly accepted by a pregnant woman in a situation of some risk. In the studies examined, this has actually occurred in only a very small percentage of cases, but note should be taken of this possible source of bias.

Thirdly, in designing the present study, we decided upon self-implementation of the intervention procedure, rather than calling upon an expert in acupuncture to apply it. The advantages of the latter approach are obvious: the correct implementation of the technique is guaranteed, it can be suited to the characteristics of each subject and it is possible to detect possible intermediate effects of the procedure, among other possibilities. However, the multicentre nature of this study means that such an approach is impractical. Thanks to the simplicity of the procedure, it can be learned rapidly by persons who are sufficiently motivated (which is indeed the case) and, in addition, with a view to its possible generalized implementation, if obvious benefits are thus gained, the design used is more appropriate to the reality of the actual public healthcare system.

Finally, it should be noted that we are investigating a desirable final effect (foetal version) but not the mechanisms by which it is achieved. Other studies have included parameters related to possible increases in foetal movements or to secondary neurohumoral changes following the application of moxa to the *Zhiyin *BL67 point. This is not the case or the aim of the present study; we believe such an investigation would require an individualized study design, distinct from that proposed in this paper.

## Competing interests

The authors declare that they have no competing interests.

## Authors' contributions

JV conceived the study, designed the study protocol, sought funding and ethical approval and wrote the manuscript. JMA, MB and CR contributed to the research design. All authors read, made critical revisions and approved the final manuscript.

## Pre-publication history

The pre-publication history for this paper can be accessed here:



## References

[B1] Westgren M, Edvall H, Nordstrom L, Svalenius E, Ranstam J (1985). Spontaneous cephalic version of breech presentation in the last trimester. Br J Obstet Gynaecol.

[B2] Cruikshank DP (1986). Breech presentation. Clin Obstet Gynecol.

[B3] Acien P (1995). Breech presentation in Spain, 1992: a collaborative study. Eur J Obstet Gynecol Reprod Biol.

[B4] Melchor JC, Fabre E (1999). Epidemiología de la presentación podálica. Manual del parto y puerperio patológicos Grupo de trabajo sobre asistencia al parto y puerperio patológicos Sección de Medicina Perinatal de la Sociedad Española de Ginecología y Obstetricia.

[B5] Cheng M, Hannah M (1993). Breech delivery at term: a critical review of the literature. Obstet Gynecol.

[B6] Danielian PJ, Wang J, Hall MH (1996). Long-term outcome by method of delivery of fetuses in breech presentation at term: population based follow up. BMJ.

[B7] Hannah ME, Hannah WJ, Hewson SA, Hodnett ED, Saigal S, Willan AR (2000). Planned caesarean section versus planned vaginal birth for breech presentation at term: a randomised multicentre trial. Term Breech Trial Collaborative Group. Lancet.

[B8] Hutton EK, Hofmeyr GJ (2006). External cephalic version for breech presentation before term. Cochrane Database Syst Rev.

[B9] Rietberg CC, Elferink-Stinkens PM, Brand R, van Loon AJ, Van Hemel OJ, Visser GH (2003). Term breech presentation in The Netherlands from 1995 to 1999: mortality and morbidity in relation to the mode of delivery of 33824 infants. BJOG.

[B10] Moldin P, Hokegard KH, Nielsen TF (1984). Cesarean section and maternal mortality in Sweden 1973–1979. Acta Obstet Gynecol Scand.

[B11] Minkoff H, Chervenak FA (2003). Elective primary cesarean delivery. N Engl J Med.

[B12] Liu GW (1996). Clinical Acupuncture & Moxibustion.

[B13] Hou JL (1995). Acupuncture and Moxibustion Therapy in Gynecology and Obstetrics.

[B14] Neri I, De PV, Venturini P, Facchinetti F (2007). Effects of Three Different Stimulations (Acupuncture, Moxibustion, Acupuncture Plus Moxibustion) of BL.67 Acupoint at Small Toe on Fetal Behavior of Breech Presentation. Am J Chin Med.

[B15] Coyle ME, Smith CA, Peat B (2005). Cephalic version by moxibustion for breech presentation. Cochrane Database Syst Rev.

[B16] Kanakura Y, Kometani K, Nagata T, Niwa K, Kamatsuki H, Shinzato Y, Tokunaga Y (2001). Moxibustion treatment of breech presentation. Am J Chin Med.

[B17] Neri I, Ternelli G, Facchinetti F, Volpe A (2000). Cardiotocography analysis during the BL67 acupoint stimulus for breech presentation. Giornale Italiano di Riflessoterapia ed Agopuntura.

[B18] Jadad AR, Moore RA, Carroll D, Jenkinson C, Reynolds DJ, Gavaghan DJ, McQuay HJ (1996). Assessing the quality of reports of randomized clinical trials: is blinding necessary?. Control Clin Trials.

